# Retraction: Akt Regulates Drug-Induced Cell Death through Bcl-w Downregulation

**DOI:** 10.1371/journal.pone.0267621

**Published:** 2022-04-20

**Authors:** The PLOS ONE Editors

Following the publication of this article [1] and Expression of Concern [2], The Ohio State University informed PLOS of the outcome of an investigation into a figure in this article.

The investigation by The Ohio State University concluded that western blot images were falsified in Figure 1B in this article [1] and/or Figure 1G in [3]. The investigation concluded:

- The β-actin panel in Figure 1B of this article [1] reports the same data as lanes 1 and 2 of the β-actin panel in Figure 1G of [3], despite representing different experimental conditions.
- Lanes 1, 2 of the Akt panel in Figure 1B of this article [1] report the same data as lanes 2, 3 of the β-actin panel in Figure 1G of [3], despite representing different experimental conditions.

Concerns with Figure 1B had previously been noted in the Expression of Concern [2] and an Editor’s Comment posted on [1] by the *PLOS ONE* Editors on 7^th^ Feb 2020. The corresponding author previously informed the journal that the original blots for Figure 1B were no longer available, and that the images in Figure 1B of [1] and Figure 1G of [3] were not the same.

The information provided by The Ohio State University supports concerns raised previously regarding the reliability and reuse of image data in Figure 1B. Therefore, in accordance with PLOS policy and COPE retraction guidelines, the *PLOS ONE* Editors retract this article.

In response to this retraction decision, the corresponding author and first author re-iterated that they do not have raw data underlying this figure, that institutional policy required data to be retained for only five years following publication, and that the investigation by The Ohio State University is being contested.

MG and GC did not agree with the retraction. All other authors either did not respond directly or could not be reached.

## References

[1] Garofalo M, Quintavalle C, Zanca C, De Rienzo A, Romano G, Acunzo M, et al. (2008) Akt Regulates Drug-Induced Cell Death through Bcl-w Downregulation. PLoS ONE 3(12): e4070. doi: 10.1371/journal.pone.0004070 19114998 PMC2603590

[2] The *PLOS ONE* Editors (2019) Expression of Concern: Akt Regulates Drug-Induced Cell Death through Bcl-w Downregulation. PLoS ONE 14(3): e0213701. doi: 10.1371/journal.pone.0213701 30889206 PMC6424394

[3] Garofalo M, Di Leva G, Romano G, Nuovo G, Suh SS, Ngankeu A, Taccioli C, Pichiorri F, Alder H, Secchiero P, Gasparini P, Gonelli A, Costinean S, Acunzo M, Condorelli G, Croce CM. miR-221&222 regulate TRAIL resistance and enhance tumorigenicity through PTEN and TIMP3 downregulation. Cancer Cell. 2009 Dec 8;16(6):498–509. doi: 10.1016/j.ccr.2009.10.014 19962668 PMC2796583

